# Evaluation of the immunogenicity and efficacy of BCG and MTBVAC vaccines using a natural transmission model of tuberculosis

**DOI:** 10.1186/s13567-019-0702-7

**Published:** 2019-10-15

**Authors:** Alvaro Roy, Irene Tomé, Beatriz Romero, Víctor Lorente-Leal, José A. Infantes-Lorenzo, Mercedes Domínguez, Carlos Martín, Nacho Aguiló, Eugenia Puentes, Esteban Rodríguez, Lucía de Juan, María A. Risalde, Christian Gortázar, Lucas Domínguez, Javier Bezos

**Affiliations:** 1 BIOFABRI S.L., Porriño, Pontevedra Spain; 2 VISAVET Health Surveillance Centre, Universidad Complutense de Madrid, Madrid, Spain; 3 Servicio de Inmunología Microbiana, Centro Nacional de Microbiología, Instituto de Investigación Carlos III, Majadahonda, Madrid, Spain; 4 Grupo de Genética de Micobacterias, Departamento de Microbiología y Medicina Preventiva, Facultad de Medicina, Universidad de Zaragoza, Zaragoza, Spain; 5 CIBER Enfermedades Respiratorias, Instituto de Salud Carlos III, Madrid, Spain; 6 Servicio de Microbiología, Hospital Universitario Miguel Servet, IIS Aragón, Zaragoza, Spain; 7 Dpto. de Sanidad Animal, Facultad de Veterinaria, Universidad Complutense de Madrid, Madrid, Spain; 8 Dpto. de Anatomía y Anatomía Patológica Comparadas, Universidad de Córdoba, Córdoba, Spain; 9 Infectious Diseases Unit, Instituto Maimonides de Investigación Biomédica de Córdoba (IMIBIC), Hospital Universitario Reina Sofía de Córdoba, Universidad de Córdoba, Córdoba, Spain; 10 SaBio (Health and Biotechnology), Instituto de Investigación en Recursos Cinegéticos IREC (CSIC-UCLM), Ciudad Real, Spain

## Abstract

Effective vaccines against tuberculosis (TB) are needed in order to prevent TB transmission in human and animal populations. Evaluation of TB vaccines may be facilitated by using reliable animal models that mimic host pathophysiology and natural transmission of the disease as closely as possible. In this study, we evaluated the immunogenicity and efficacy of two attenuated vaccines, BCG and MTBVAC, after each was given to 17 goats (2 months old) and then exposed for 9 months to goats infected with *M. caprae*. In general, MTBVAC-vaccinated goats showed higher interferon-gamma release than BCG vaccinated goats in response to bovine protein purified derivative and ESAT-6/CFP-10 antigens and the response was significantly higher than that observed in the control group until challenge. All animals showed lesions consistent with TB at the end of the study. Goats that received either vaccine showed significantly lower scores for pulmonary lymph nodes and total lesions than unvaccinated controls. Both MTBVAC and BCG vaccines proved to be immunogenic and effective in reducing severity of TB pathology caused by *M. caprae*. Our model system of natural TB transmission may be useful for evaluating and optimizing vaccines.

## Introduction

Tuberculosis (TB) is a multi-host zoonotic disease that affects a wide range of domestic and wild animals. TB in animals is caused by members of the *Mycobacterium tuberculosis* complex (MTBC), principally *M. bovis* and *M. caprae*. Consumption of raw milk and close contact with infected animals are the most common routes of transmission to humans [1]. TB causes public health problems as well as economic losses for the livestock sector, which arise because of production losses and trade restrictions. It is, therefore, of paramount importance to prevent the development of advanced lesions that can result in an increased aerosol transmission between animals or between animals and humans, such as farmers, abattoir workers or veterinarians.

Policies to check for TB in animals focus on testing and slaughtering reactor cattle [2, 3]. However, routine diagnostic tests and compensation for slaughter are unavailable in many countries, making cost-effective alternatives such as vaccination of great interest. Vaccination should target not only immediate animal hosts but also other domestic and wild hosts that can help maintain the disease [4] such as goats or wild animals, which help maintain TB in cattle [5]. Combining vaccination with eradication programs requires TB diagnostic tests that can differentiate between infected and vaccinated animals (DIVA strategy) and that have a sensitivity as high as the current official diagnostic tests based on protein purified derivative (PPD) [6]. At present, cattle vaccination is forbidden in the European Union (Chapter III, Article 13, Council Directive 78/52/EEC), and only the Bacille Calmette–Guérin (BCG) vaccine is licensed for use in badgers in the UK (Marketing authorisation Vm 03326/4021).

In humans, TB is caused mainly by *M. tuberculosis* and it is still the leading cause of death from a single infectious agent. Moreover, drug-resistant TB is an increasing threat [7]. Thus, effective vaccines to prevent TB transmission are urgently needed. BCG is the only vaccine licensed for use in humans. It can protect against meningeal and disseminated (extra-pulmonary) TB in children [8]. However, it shows variable efficacy in preventing pulmonary TB in adults [8, 9]. A promising potential alternative to BCG is MTBVAC, another attenuated *M. tuberculosis* vaccine that is in phase II clinical trials in neonates (clinical trial identifier: NCT035336117) and adolescents (NCT02933281). Of the 13 TB vaccines currently in clinical trials, only MTBVAC contains attenuated *M. tuberculosis* [7]. MTBVAC is based on two independent genetic deletions in the genes *phoP* and *fadD26,* which encode two major virulence factors and conserve genetic regions that encode important and immunodominant antigens absent from BCG [10]. In previous studies, the SO2 prototype vaccine (including only the *phoP* deletion) and subsequent MTBVAC vaccine proved to have greater immunogenicity and efficacy than BCG in mice [11–13], guinea pigs [11, 14] and rhesus macaques [15]. SO2 conferred partial protection on goats naturally exposed to *M. bovis* and *M. caprae* but no results of efficacy using MTBVAC have been reported [16]. Goats are a suitable model for TB studies and it has been used in previous vaccination studies [17].

The main objective of the present study was to evaluate, for the first time, the immunogenicity and protective efficacy of MTBVAC in goats naturally exposed to *M. caprae.* Of all species in the MTBC, *M. caprae* is the most frequent in goats. This study aimed to examine cellular and humoral responses triggered by MTBVAC in goats, as well as the protection conferred by an attenuated *M. tuberculosis* vaccine. The study also assessed possible interference from the MTBVAC vaccine in current TB diagnostic tests in animals using PPDs, ESAT-6, CFP-10 or Rv3615c as antigens.

## Materials and methods

### Experimental design

**Figure 1:**
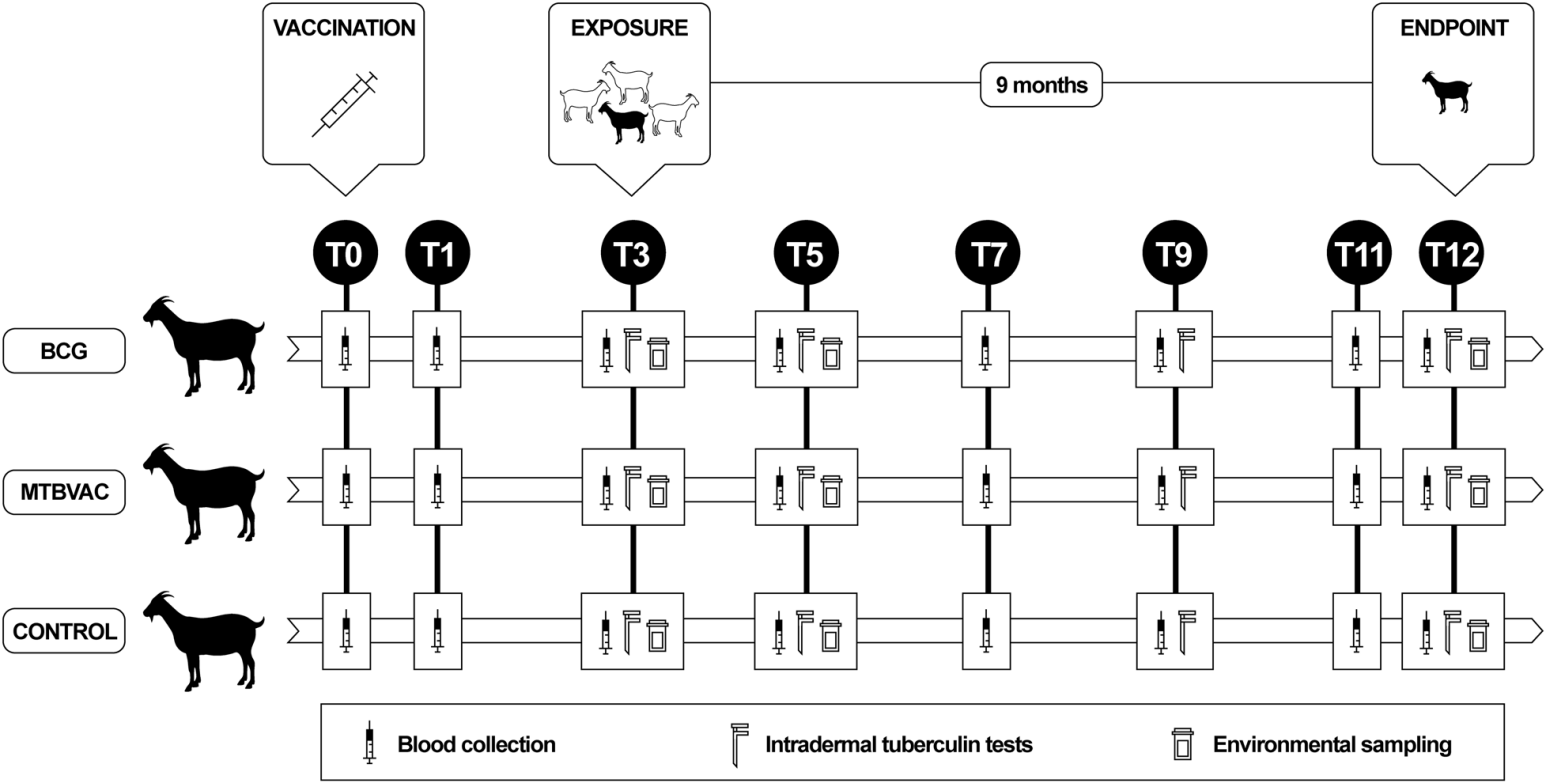
**Experimental design.** Black goat silhouettes represent receptor goats (vaccinated and control groups), and white goat silhouettes represents the donor goats infected with *M. caprae.*

Fifty-one Murciano–Granadina goat kids (8 weeks old) were selected from a farm in Spain with no history of TB that imposed strict biosecurity measures and raised kids artificially, producing animals of high genetic value. All goats were confirmed to be TB-negative using a commercial interferon-gamma release assay (IGRA; Bovigam TB kit, Thermo Fisher Scientific, Waltham, USA) based on criteria recommended by the Spanish TB eradication program (Ministry of Agriculture, Fisheries and Food) for cattle and goats (see IGRA section below). The animals were distributed into three groups (Figure 1): BCG (*n* = 17), MTBVAC (*n* = 17) and control (*n* = 17).

The first two animal groups received, at 2 months of age, MTBVAC vaccine (0.1 mL, 5 × 10^5^ colony forming units; lot number 143072, Biofabri SL, Porriño, Spain) or BCG Danish SSI 1331 (2–8 × 10^5^ colony-forming units; lot number L389336B, Statens Serum Institute, Copenhagen, Denmark). Vaccines were obtained as freeze-dried preparations and were reconstituted according to the manufacturers’ instructions in Sauton’s medium (BCG) or MTBVAC diluent (Biofabri, Porriño, Spain). Vaccines were administered subcutaneously into the medial area of the left-hand side of the neck using a needle 16 mm long. Control goat kids remained unvaccinated.

At 3 months post-vaccination, all three groups of goat kids were exposed to 30 reactor goats from an infected herd. The reactor goats were TB-positive based on IGRA and single intradermal tuberculin (SIT) tests, and the donor farm was confirmed to have TB caused by *M. caprae* spoligotype SB0157. Vaccinated and control goat kids cohabited with the reactor goats for 9 months in a biosafety facility. Animal handling, testing and sampling were performed by qualified veterinarians in accordance with European (86/609/CEE) and Spanish (RD 53/2013) legislation. All procedures were authorised by an institutional ethical committee and approved by the local authorities (PROEX: 411/15; Comunidad de Madrid).

### IGRA

Blood samples were collected immediately prior to vaccination (T0, where T# refers to how many months after vaccination), T1, T3 (exposure), T5, T7, T9, T11, and T12 (end point) (Table 1). Heparinised blood samples were stimulated as described [18] with bovine protein purified derivative (PPD-B) and avian PPD (PPD-A) (CZ Vaccines, Porriño, Spain) at a final concentration of 20 µg/mL, along with the peptide cocktail ESAT6/CFP10 (E/C) and peptide Rv3615c (provided by the Animal and Plant Health Agency, Addlestone, UK); both peptides were given at a final concentration of 5 μg/mL. IFN-γ levels in plasma were measured using a commercial IGRA (Bovigam TB kit). Animals were considered to be positive when the optical density (OD) of a sample stimulated with PPD-B, after subtracting the OD of phosphate-buffered saline (PBS), was ≥ 0.05 and greater than the OD of the sample stimulated with PPD-A. A less stringent threshold of 0.1 was applied when samples were stimulated with PPD-A or the E/C peptide cocktail and peptide Rv3615c. Results for the E/C peptide cocktail and peptide Rv3615c were interpreted separately from each other and without taking into account the OD for samples stimulated with PPD-A [16, 18].

**Table 1:** **Number of positive reactors in each experimental group using different diagnostic tests and antigens**

Test	Antigen	Group	T0^a^	T1	T3^b^	T5	T7	T9	T11	T12
IGRA	PPD-B cut-off 0.05	Control	0/17	0/17	0/17	2/17	9/15	12/14	10/12	10/12
		BCG	0/17	2/17	6/17	0/17	3/15	7/14	10/14	8/14
		MTBVAC	0/17	14/17	7/17	1/16	5/15	11/13	11/13	12/13
	PPD-B cut-off 0.1	Control	0/17	0/17	0/17	2/17	8/15	12/14	10/12	10/12
		BCG	0/17	2/17	5/17	0/17	1/15	3/14	7/14	6/14
		MTBVAC	0/17	12/17	4/17	0/16	3/15	8/13	6/13	9/13
	E/C^c^	Control	0/17	0/17	NA	2/17	5/15	11/14	6/12	7/12
		BCG	0/17	0/17	NA	0/17	0/15	0/14	2/14	1/14
		MTBVAC	0/17	3/17	NA	1/16	0/15	4/13	1/13	5/13
	Rv3615^c^	Control	0/17	0/17	NA	1/17	3/15	8/14	3/12	3/12
		BCG	0/17	0/17	NA	0/17	0/15	0/14	0/14	0/14
		MTBVAC	0/17	1/17	NA	0/16	0/15	1/13	1/13	3/13
SIT test	PPD-B	Control	NA	NA	1/17	2/17	NA	11/14	NA	11/12
		BCG	NA	NA	15/17	4/17	NA	2/14	NA	11/14
		MTBVAC	NA	NA	15/17	1/16	NA	8/13	NA	12/13
	E/C	Control	NA	NA	NA	2/17	NA	9/14	NA	6/12
		BCG	NA	NA	NA	1/17	NA	2/14	NA	2/14
		MTBVAC	NA	NA	NA	0/16	NA	5/13	NA	5/13
SCIT test	PPD-B and PPD-A	Control	NA	NA	0/17	2/17	NA	7/14	NA	10/12
		BCG	NA	NA	10/17	1/17	NA	1/14	NA	8/14
		MTBVAC	NA	NA	11/17	0/16	NA	6/13	NA	9/13
P22_ELISA (E% 100)	P22 and PPD-A	Control	0/17	0/17	0/17	0/17	3/15	2/14	8/12	6/12
		BCG	0/17	0/17	0/17	3/17	5/15	2/14	11/14	13/14
		MTBVAC	0/17	1/17	0/17	3/16	8/15	1/13	12/13	10/13
P22_ELISA (E% 150)	P22 and PPD-A	Control	0/17	0/17	0/17	0/17	2/15	2/14	4/12	6/12
		BCG	0/17	0/17	0/17	2/17	2/15	1/14	4/14	5/14
		MTBVAC	0/17	1/17	0/17	0/16	3/15	0/13	4/13	8/13

E%, ELISA percentage for interpretation and cut-off point; E/C, cocktail of ESAT-6/CFP-10; IGRA, interferon-gamma release assay; NA, not available; PPD-B, bovine purified protein derivative; PPD-A, avian purified protein derivative; SIT, single intradermal tuberculin test; SCIT, single comparative intradermal tuberculin test.

^a^Vaccination (time in months).

^b^Exposure to infected animals.

^c^Cut-off 0.1.

### Intradermal tuberculin tests

Vaccinated and control goats were subjected to a SIT test and a single comparative intradermal tuberculin (SCIT) test at T3 (exposure), T5, T9 and T12. Both tests were performed according to Council Directive 64/432/EEC and Royal Decree RD2611/1996. PPD-B and PPD-A (0.1 mL; CZ Vaccines, Porriño, Spain) were inoculated on the left-medial or right-medial side of the neck, respectively. The test was interpreted for all animals by the same veterinarian 72 h later. The SIT test was considered positive when skin fold thickness increased by ≥ 4 mm or clinical signs (exudation, oedema or necrosis) were detected. The SCIT test was considered positive when the bovine reaction was larger than the avian reaction by more than 4 mm or clinical signs were observed at the bovine site. The animals were inoculated with a cocktail of ESAT-6 and CFP-10 proteins (E/C, 100 μg/mL; Lionex, Braunschweig, Germany) at T5, T9 and T12. The intradermal E/C test results were interpreted in the same way as the SIT test results.

### Serology

An in-house competitive P22 ELISA, which measures immunoreactivity against a protein P22 affinity-purified from bovine PPD (CZ Vaccines, Porriño, Spain), was performed at T0, T1, T3, T5, T7, T9, T11 and T12 as described elsewhere [19]. Briefly, assay plates were coated overnight at 4 °C with 50 μL of P22 at 10 μg/mL, blocked with 5% skimmed milk powder solution in phosphate-buffered saline (PBS) for 1 h at room temperature, and washed three times with PBS containing 0.05% Tween-20 (PBST). Sera were diluted 1:100 in skim milk and supplemented with avian PPD at 150 μg/mL, then added in duplicate the wells. Plates were incubated for 60 min at 37 °C. Horseradish peroxidase-conjugated rabbit anti-sheep IgG (H + L, diluted 1:2000, 100 μL; SouthernBiotech, Birmingham, USA) was added, and plates were incubated for 30 min at room temperature. Plates were washed five times with PBST, and color was developed by adding 100 μL of o-phenylenediaminedihydrochloride substrate (FAST OPD, Sigma-Aldrich, St. Louis, USA) and incubated for 15 min in darkness at room temperature. The reaction was stopped with 50 μL of 3 N H_2_SO_4_. OD at 492 nm was measured with an ELISA reader. Negative control serum was obtained from TB-free goats that were negative for MTBC culture; positive control serum was obtained from goats positive for MTBC culture. Positive and negative controls were included in every plate in quadruplicate. ELISA results were expressed as an ELISA percentage (E%) = [mean sample OD/(2 ×  mean of negative control OD)] ×  100. The cut-off value was defined as the ratio of the mean sample OD to the double of the mean OD of the negative control. Serum samples with E% values greater than 100 were considered positive. A less stringent cut-off of E% ≥ 150 was also applied [19].

### Environmental sampling

The environmental circulation of mycobacteria within the herd and their presence on the body surface of the vaccinated and control groups were evaluated by scrubbing the animals’ skin at T3, T5 and T12 with pre-hydrated sponges containing 15 mL of a liquid solution (patent pending). Animals were scrubbed 10 times on both sides of the dorsolateral thorax and abdomen. The fluid was then recovered from the sponge, re-diluted by addition of 10 mL and centrifuged at 1500 *g* for 10 min. The DNA was extracted using a DNeasy^®^ Blood & Tissue kit (Qiagen, Hilden, Germany), then used as template in quantitative PCR amplification of the IS*6110* sequence [20].

### Gross lesions and histopathology

At 9 months after exposure and 12 months after vaccination (T12), the receptor and donor animals were sedated by means of an intravenous injection of xylazine at 10 mg/50 kg (2% Xilagesic, Calier SA, Barcelona, Spain), and then euthanized with an intravenous injection of T-61 (MSD Animal Health, Salamanca, Spain). The thoracic perimeter was measured (in cm), and differences among groups as well as the association with thorax pathology were analysed. Gross lesions on all organs were systematically examined using two semi-quantitative systems, one for the lungs and another for the lymph nodes (LNs) and remaining organs. Gross lesions in the lung lobes were categorised in five groups according to the percentage of the lobe affected: 0 or no evident TB-compatible lesions (TBCL); 1, under 25% of the lung lobe affected; 2, 25–50%; 3, 50–75%; and 4, > 75%. One extra point was given to animals that had pleural adhesions. The total lung score was the sum of the scores for each lung lobe (left apical, left diaphragmatic, right apical, right cardiac, right accessory and right diaphragmatic). The size and number of lesions were scored across six categories as described [21] in retropharyngeal LNs, pulmonary LNs (left and right tracheobronchial, and mediastinal), hepatic LN, ileocecal LN and mesenteric LNs. Lesions were also scored in other organs containing TBCLs. The following scoring system was applied: 0, no visible lesions; 1, no gross lesions but lesions apparent on slicing; 2, ≤ 5 gross lesions < 10 mm in diameter; 3, ≥ 6 gross lesions < 10 mm in diameter or a single gross lesion > 10 mm in diameter; 4, > 1 distinct gross lesion > 10 mm in diameter; 5, coalescing gross lesions. The pulmonary LN score was the sum of the scores for the left and right tracheobronchial as well as mediastinal LNs. The pulmonary LN score and extra-pulmonary LN scores and total lung scores were added up to determine the total score per animal. The personnel in charge of the necropsies were blinded to the identity of the group of vaccinated animals examined, and the same assessor scored all animals in order to ensure scoring consistency.

Tissue samples were fixed in 10% phosphate-buffered formalin for 48 h before being embedded in paraffin wax. Four-micron sections were cut and stained with hematoxylin and eosin (H&E). Histopathology analysis was carried out by examining three microscopic fields (40×) from a section of 1 × 1 cm of cranial and caudal mediastinal, left and right tracheobronchial LNs and lung in order to assess the quantity of multinucleated giant cells (MNGCs) and the number and stage of the granulomas (I–IV), where stage I is the initial; stage II, solid; stage III, minimal necrosis; and stage IV, necrosis and mineralisation [22]. The presence of small satellite granulomas surrounding a central lesion [23] was also recorded.

### Bacteriology

The tissue samples comprised head and thorax tissues obtained from the retropharyngeal, mediastinal and bronchial LNs and lungs. The samples were decontaminated with 0.37% hexadecylpyridinium chloride [24], and then cultured on Coletsos and 0.2% (w/v) pyruvate-enriched Löwenstein–Jensen media (Difco, Madrid, Spain). Isolates were identified as MTBC using conventional PCR and/or DVR-spoligotyping [25]. The head and thorax tissue samples were cultured in parallel on Columbia Agar media plates with 5% of sheep’s blood (BioMèrieux, Madrid, Spain) for the isolation of *Corynebacterium pseudotuberculosis* as described [16].

Bacterial DNA was quantified in 2-g pooled samples of respiratory LNs and lungs after 28 days of culturing in liquid medium (Bactec MGIT 960, Becton–Dickinson). DNA was extracted from 1.5 mL of liquid medium from positive samples. The medium was centrifuged at 9000 *g* for 5 min, the supernatant was removed and the pellet was washed with sterile distilled H_2_O, centrifuged again, suspended in 200 μL of water, and heat-inactivated. Purity and concentration of DNA samples were measured using a NanoDrop 2000 spectophotometre (Thermo Fisher Scientific, Waltham, USA). Bacterial growth was quantified absolutely using qPCR targeting the *mpb70* gene [26]. The standard DNA curve was generated using DNA extracted from a *M. bovis* AN5 culture by phenol:chloroform:isoamyl alcohol. DNA concentration was adjusted to 1.2 ng/μL (approximately 2.53 × 10^5^ copies/μL) and 12 fg/μL (2.53 copies/μL) using a Qubit 4 fluorometer (Thermo Fisher Scientific). The number of copies of the *mpb 70* gene was defined as equal to the number of bacteria, since MTBC species contain only one copy of this gene [26].

### Statistical analyses

All tests were carried out using SPSS 25 (IBM, New York, USA), and a *p* value of 0.05 was defined as the cut-off for statistical significance. The confidence intervals for proportions were calculated according to Wilson’s 95% intervals. Normality of quantitative values was assessed using the Kolmogorov–Smirnov test. Fisher’s exact test was used to compare the proportions of positive test results between groups, as well as to assess homogeneity in the results for TBCL presence or absence and for infection prevalence. The Kruskal–Wallis test was used to compare quantitative results between groups, such as skin fold thickness, IFN-γ level, P22 ELISA OD, thoracic perimeter and lesion score; this test was followed by pairwise tests for multiple comparisons of mean rank sums after Bonferroni correction of the *p* value. Quantitative values were compared between different time points using the Wilcoxon signed-rank test. The Spearman’s rank correlation coefficient (rho) was used to assess relationships among IFN-γ levels after stimulation with E/C and PPD-B, the increase in skin fold thickness, the thoracic perimeter or P22 ELISA OD and the lesion score.

## Results

### Clinical signs and follow-up

No clinical signs or adverse reactions were observed at the site of vaccine inoculation in any of the vaccinated animals. At the final time point, thoracic perimeter did not differ significantly among the groups (*p* = 0.157; median = 65.3 cm, interquartile range (IQR) 63.6–66.8; BCG, median = 67.8 cm, IQR 63.1–72.5; MTBVAC, median = 66.5 cm, IQR 63.3–68.5).

Three animals in the BCG group, four in the MTBVAC group and five in the control group died between T5 and T11, corresponding to between 2 and 8 months post-exposure. One goat without TBCL from the MTBVAC group and three control goats with TBCL were culture-positive. None of the 8 remaining fatalities had either TBCL or positive culture. All 12 animals were excluded from the post-mortem analysis because the presence and severity of their lesions were not comparable to those of the animals slaughtered at the end point.

### Immune response to vaccination and natural exposure

**Figure 2:**
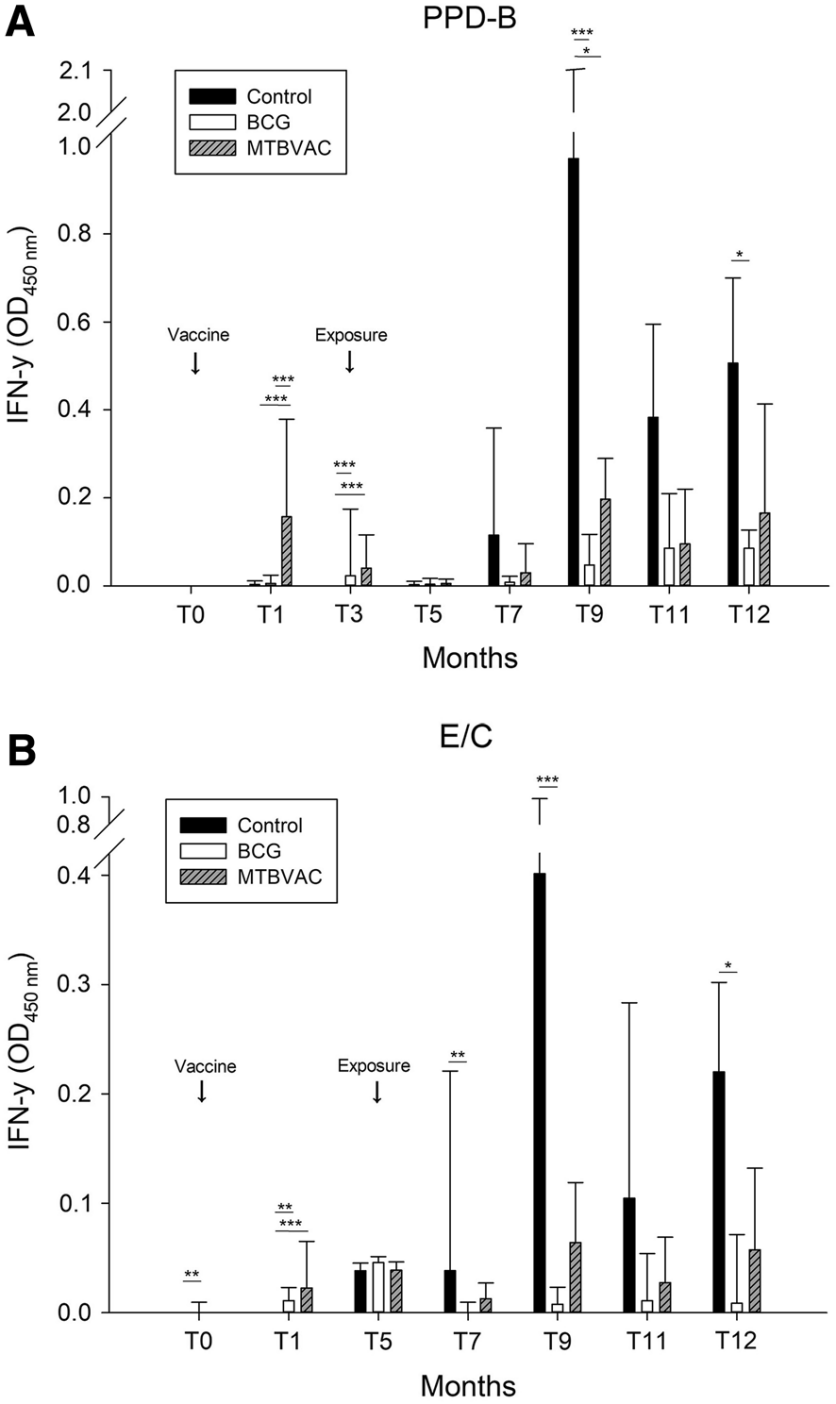
**Median and interquartile range of IFN-γ (OD**_**450nm**_**) in blood samples after stimulation with PPD-B (A) or E/C cocktail (B) in each animal group at different time points during the study.** ***p < 0.001; **p < 0.01; *p < 0.05.

The numbers of reactors to the cellular and antibody-based tests (IGRA, SIT, SCIT, P22 ELISA) are summarised in Table 1. At T1, the MTBVAC group had a significantly greater number of reactors to the IGRA based on PPD-B (using 0.05 cut-off, 82.4%, 95% CI 59–93.8; using 0.1 cut-off, 70.6%, 95% CI 46.8–86.7) than the BCG and control groups (*p *< 0.001). From T1 to T3, the percentage of IGRA-positive animals (0.05 cut-off) decreased in the MTBVAC group but increased in the BCG group, and both percentages remained significantly higher than in the control group (*p* = 0.007 and *p* = 0.018, respectively). In all groups, IFN-γ response to PPD-B started to increase steadily from T7 to T12, corresponding to 4 months after exposure until the end of the experiment. IFN-γ levels peaked at T9 in the MTBVAC and control groups, yet levels in the MTBVAC and BCG groups were significantly lower than in the control group (Figure 2A; *p* = 0.049 and *p *< 0.001, respectively). At the end of the study, the BCG group, but not the MTBVAC group, showed significantly lower IFN-γ levels than the control group (Figure 2A; *p* = 0.020).

Based on a 0.1 cut-off, three MTBVAC animals were positive for E/C at T1 (specificity = 82.35%, 95% CI 58.97–93.81). At T7, neither vaccinated group contained animals positive for the IGRA based on E/C, a rate significantly lower than in the control group (Table 1; both *p* = 0.042). However, by the final time point before necropsies, the MTBVAC group no longer differed significantly from the control group, while the BCG group did (Table 1; *p* = 0.009). After 4, 6 and 9 months in contact with infected donors (T7, T9 and T12), the BCG group, but not the MTBVAC group, showed significantly lower IFN-γ response to E/C than the control group (Figure 2B; T7, *p* = 0.001; T9, *p *< 0.001; T12, *p *= 0.010). At T12, IFN-γ levels in response to E/C when considering all the groups together showed a moderate, positive correlation with total lesion score (rho = 0.496, *p *< 0.01).

The proportion of IGRA-positive reactors among the infected ones in the control group at T12 increased from 58.3% (95% CI 32–80.7) when only the E/C cocktail was used to 75% (95% CI 46.8–91.1) when the cocktail was used together with Rv3615c peptide. One additional animal was positive when PPD-B was used (83.3%, 95% CI 55.2–95.3). Rv3615c did not allow detection of any infected BCG animals, and it allowed detection of some infected MTBVAC animals (Table 1).

Before exposure (T3), both vaccinated groups showed higher reactivity on the SIT and SCIT tests using PPDs than the control group did (*p *< 0.001). At this time point, the increase in skin fold thickness after PPD-B inoculation was higher for the two vaccinated groups than for the control group (both *p *< 0.001). Similar results were observed for T5 (*p* = 0.038 and *p* = 0.001, respectively). All three groups showed maximal increase in skin fold thickness at T9 as observed in the IGRA levels using PPD-B and E/C (Figure 2A and B), and at this time point, BCG goats showed a significantly smaller increase in skin fold thickness than control goats (*p* = 0.002).

**Figure 3:**
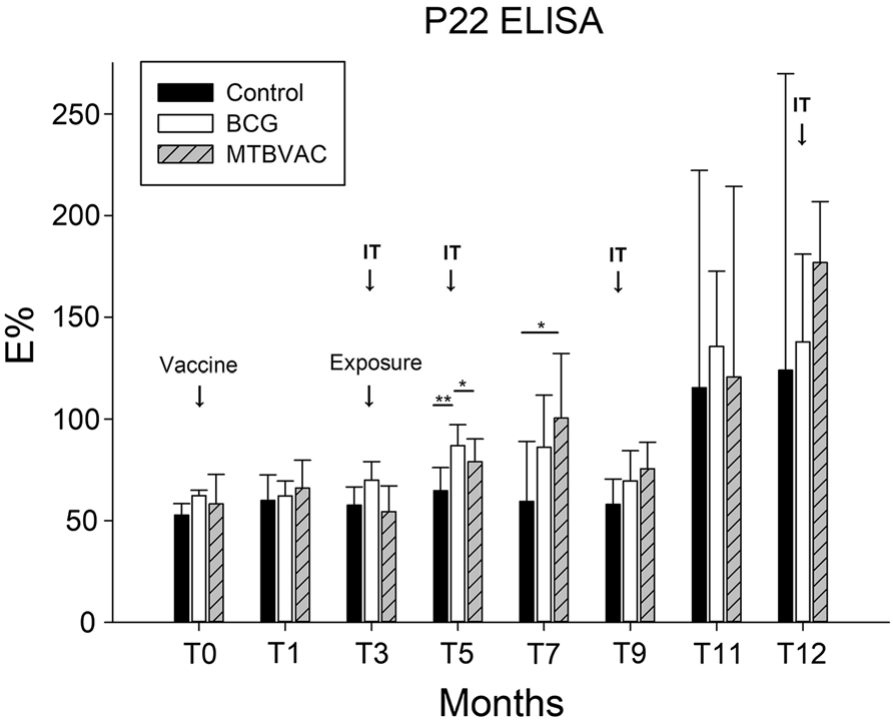
**Median and interquartile range of the ELISA percentage (E%) observed in the P22 ELISA in each group at different time points during the study.** Intradermal tests (ITs) were performed at T3, T5, T9 and T12 (black arrows). ***p < 0.001; **p < 0.01; *p < 0.05.

Antibody levels against P22 increased over time from T3 to T12 (except T9) in all groups (Figure 3). Only one goat from the MTBVAC group had an antibody titre over the cut-off point after vaccination (T1) and before exposure. At T5, the median E% value was higher in the BCG group than in the control group (*p *= 0.003) and MTBVAC group (*p* = 0.043). At T7, the median E% was significantly higher in the MTBVAC group than in the control group (*p* = 0.032). At the end point (T12), only 50% (95% CI 25.4–74.6) of control goats were positive in the P22 ELISA according to both cut-offs (Table 1). However, 13/14 of the BCG-vaccinated goats and 10/13 of MTBVAC-vaccinated goats were positive according to the stringent cut-off. No correlation was observed between the E% and the total lesion score at T12 when considering all the groups together (rho = − 0.31, *p* = 0.851) or when considering only the control group (rho = 0.218, *p* = 0.494). The intradermal tests may have affected the antibody levels in all groups, since E% values for the intradermal tests increased significantly from T3 to T5 in all the groups (BCG, *p* = 0.002; MTBVAC, *p* = 0.003; control, *p* = 0.049), as well as from T9 to T11 (BCG, *p* = 0.001; MTBVAC, *p* = 0.003; control, *p* = 0.003).

### Environmental DNA

At 2 months post-exposure (T5), the rate of MTBC identification was 82% of the samples (41/50, 95% CI 69.2–90.2), without significant differences among groups (control = 13/17, BCG = 16/17, MTBVAC = 12/16). Seven months later (T12), all sponge samples were positive for MTBC (39/39, 100%, 95% CI 91.0–100).

### Post-mortem examination

**Figure 4:**
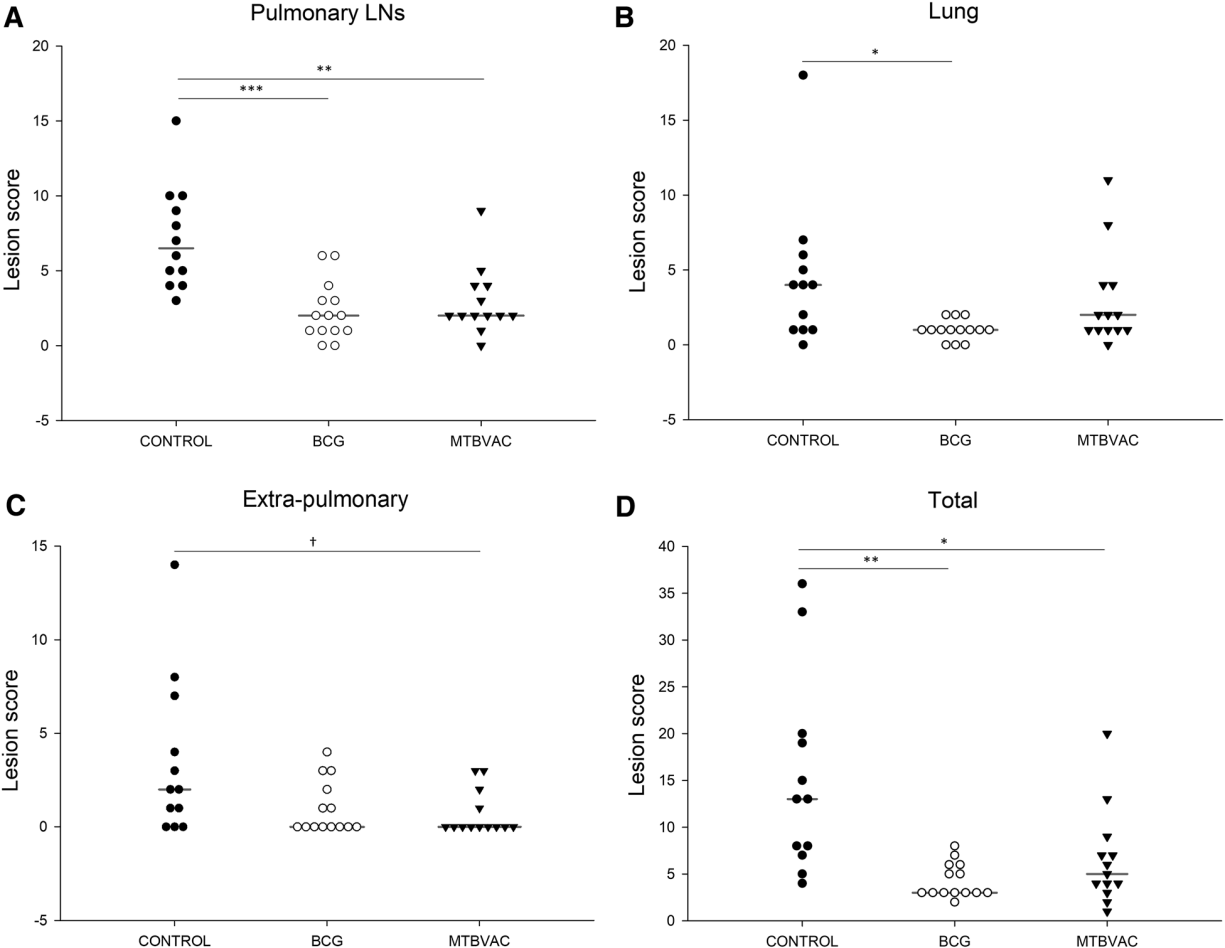
**Lesion scores in pulmonary lymph nodes (LNs) (A), lungs (B), extra-pulmonary organs (C) and in all organs examined (D) in the control, BCG and MTBVAC groups.** Horizontal lines represents median values. ***p < 0.001; **p < 0.01; *p < 0.05; ^†^*p* < 0.1.

TBCLs were observed in the lungs or pulmonary LNs of all animals. There were lesions in 93.3% (95% CI 78.7–98.2) of donor goats, all of which died during the study or were euthanized at the end point. BCG and MTBVAC-vaccinated goats showed significantly lower pulmonary LN scores than controls (*p *< 0.001 and *p* = 0.005, respectively) as well as lower total lesion scores (*p* = 0.001 and *p* = 0.032, respectively) (Figure 4A and D). The BCG group, but not the MTBVAC group, showed significantly lower lung lesion score than the control group (*p* = 0.028; Figure 4B). The BCG group, but not the MTBVAC group, also showed a significantly lower median number of affected lung lobes than the control group (Table 2). The most affected lobes in all groups were the caudal ones: gross lesions in the right caudal lobe were most prevalent in control goats (10/12) and BCG goats (5/14), while gross lesions in the left caudal lobe were most prevalent in MTBVAC goats (10/13). No extra-pulmonary lesions were observed in 8 goats vaccinated with BCG (57.1%, 95% CI 32.6–78.6) and 9 vaccinated with MTBVAC (69.2%, 95% CI 42.4–87.3), compared to only 3 controls (8.3%, 95% 1.5–35.4) (BCG, *p* = 0.130; MTBVAC, *p* = 0.047). Extra-pulmonary lesion scores in the control group were similar to those in the BCG group (*p* = 0.181) and MTBVAC group (*p* = 0.051) (Figure 4C).

**Table 2:** **Gross and histopathological analysis of TBL in lung and pulmonary lymphoid nodes**

	Control	BCG	MTBVAC
Lung			
Median no. of lobes affected	3.5 (IQR 1–4)	1 (IQR 0.8–1)*	2 (IQR 1–2.5)
Median score	4 (IQR 1–5.8)	1 (IQR 0.8–1.3)*	2 (IQR 1–4)
No. animals with TBL	10/11 (83.3%)^b^	3/14 (21.4%)*	8/13 (61.5%)
Development stage of granulomas			
I	0/11 (0%)	0/14 (0%)	0/13 (0%)
II	1/11 (9.1%)	0/14 (0%)	3/13 (23.1%)
III	2/11 (18.2%)	0/14 (0%)	0/13 (0%)
IV	7/11 (63.6%)	3/14 (21.4%)*	5/13 (38.5%)
Mean of MNGCs^a^	7 (± 8.1)	1.6 (± 3.7)*	5.1 (± 8)
No. animals with satellite granulomas	0/11 (0%)	1/14 (7.1%)	0/13 (0%)
Pulmonary LNs			
Median score	6.5 (IQR 4.3–9.8)	2 (IQR 1–3.3)*	2 (IQR 2–4)*
No. animals with TBL	11/11 (100%)^b^	8/14 (57.1%)*	10/13 (76.9%)
Development stage of granulomas			
I	0/11 (0%)	0/14 (0%)	1/13 (7.7%)
II	1/11 (9.1%)	0/14 (0%)	0/13 (0%)
III	1/11 (9.1%)	0/14 (0%)	0/13 (0%)
IV	9/11 (81.8%)	8/14 (57.1%)	9/13 (69.2%)
Mean of MNGCs	10.7 (± 6.9)	3.71 (± 5.52)*	9.1 (± 11.7)
No. animals with satellite granulomas	5/11 (45.5%)	1/14 (7.1%)	4/13 (30.8%)

IQR, interquartile range; MNGC, multinucleated giant cells; TBL, tuberculous lesion.

* Statistically significant difference with regard to the control group (*p *≤ 0.05).

^a^MNGCs per animal (mean of three microscopic fields 400×).

^b^Lung and pulmonary samples from one animal were not well fixed in formalin and could not be assessed for histopathology.

Table 2 presents the histopathology findings. Stage IV was the stage of granuloma most frequently observed in the lungs and pulmonary LNs of all groups. These lesions were characterised by a central necrosis with mineralisation surrounded by a granulomatous inflammatory response. Macrophages and epitheloid cells were aggregated around the necrotic lesions, forming Langerhans giant cells, and were significantly fewer in the lungs and pulmonary LNs of the BCG group than in the other groups (Table 2). Similar proportions of animals in all three groups showed satellite granulomas in their lungs and pulmonary LNs.

### Bacteriology

The isolation rate was 12/12 (100%, 95% CI 75.8–100) in the control group, 8/14 (57.1%, 95% CI 32.6–78.6) in the BCG group and 11/13 (84.6%, 95% CI 57.8–95.7) in the MTBVAC group (BCG, *p* = 0.017; MTBVAC, *p* = 0.48). The only spoligotype identified was *M. caprae* SB0157. Moreover, two animals from the control and MTBVAC groups were co-infected with *Corynebacterium pseudotuberculo*sis/*M. caprae*, and *C. pseudotuberculosis* alone was isolated from one goat vaccinated with BCG. The isolation rate in donor goats was 80% (95% CI 62.7–90.5); donor animals did not undergo the detailed necropsy of receptor goats.

Quantitative PCR indicated similar bacterial DNA levels in respiratory LNs and lungs across the groups. The median value was 5.2 × 10^4^ bacteria/μL (IQR, 4.2 × 10^4^–7.5 × 10^4^) in the BCG group, 1.03 × 10^5^ bacteria/μL (IQR 3.4 × 10^4^–12.8 × 10^4^) in the MTBVAC group, and 6.8 × 10^4^ bacteria/μL (IQR 4.2 × 10^4^–9.2 × 10^4^) in the control group.

## Discussion

In the present study, BCG and MTBVAC vaccines led to milder gross TB pathology under conditions of long-term exposure to *M. caprae*. MTBVAC effectively reduced the frequency of animals with extra-pulmonary TB and severity of TBCLs in pulmonary LNs, while BCG reduced the pathology severity in lungs and pulmonary LNs. For 9 months, vaccinated and control goats were in constant direct contact, via aerosols, with infected goats, and they shared feed and water points, providing a natural transmission model similar to that described for goats vaccinated with the *M. tuberculosis* SO2 strain [16]. Natural transmission models can be a particularly reliable method to evaluate vaccines, treatments and diagnostic tests for animals or humans, but they also have disadvantages, including high maintenance costs and the impossibility of knowing infection dates or exposure doses. Therefore, the development of new biomarkers correlated with disease progression would be valuable to reliably set end points in field and laboratory trials.

Environmental sampling confirmed the continuous exposure in our natural transmission model, since MTBC was detected in 82% of samples at 2 months post-exposure (T5). This innovative technique is easy to perform and useful as a surveillance tool for the analysis of environmental bacterial load and potential risk of exposure not only in the case of TB but other infections as well. The high bacterial load and persistent exposure on the farm may have predisposed all goats to develop visible TB lesions and high culture positivity. Transmission may also have been promoted by the fact that goats are the natural hosts of *M. caprae*, the SB0157 spoligotype is the most frequent *M. caprae* strain isolated from cattle in Spain [27], and SB0157 is associated with severe TB in Eurasian wild boar [28]. A similar study evaluating the SO2 prototype of MTBVAC in goats that were kept in contact with donors infected with *M. bovis* (SB0134 and SB0339) and *M. caprae* (SB0157) found that the prototype led to 63.6% lower mean total lesion scores and 89.5% lower lung lesion scores than in unvaccinated goats [16]. In the present study, we introduced animals from only one origin infected with *M. caprae* SB0157, and the MTBVAC led to 57% lower mean total lesion scores and 34.1% lower lung lesion scores than in controls. Severity of gross lesions was similar between the BCG and MTBVAC groups. In our earlier work, we found that the SO2 vaccine led to lower lesion scores and lower proportion of bacteriology isolation than BCG, although imbalance between the sizes of the BCG and SO2 groups prevented definitive conclusions [16]. The period of exposure was similar in that previous study using the SO2 vaccine and the present study but other factors could be responsible for the different reduced gross pathology observed between studies. One of the factors may be the lower ratio of donors/vaccinated goats in the SO2 study compared to the present one. Another point to take into consideration is the MTBC species and strain, since in the present study, vaccinated goats were exposed to a group of infected donors with one *M. caprae* strain throughout the study, whereas in the previous SO2 study, vaccinated goats were first exposed to infected donors with two *M. bovis* strains for 18 weeks and then to infected donors with a single *M. caprae* strain the following 22 weeks. Therefore, the virulence of the MTBC species and strains in goats might also have played an important role in the differences in lesion severity as suggested by Bezos et al. [29].

In the present study, the rate of bacteriological isolation in the thorax was lower in the BCG group than in the control group. Nevertheless, no differences were found in the quantification of bacterial DNA from the lungs and pulmonary LNs samples across all three groups. Lesion grade here did not correlate with bacterial load, in contrast to what has previously been described in goats [23, 30] and nonhuman primates [31, 32]. The higher bacterial load could also be related to the type of the lesions (cavitary vs granulomatous) [17], but we could not discriminate between different lesions in this study since samples from different tissues were pooled. Histopathological analysis revealed that most animals had Stage IV granulomas in the lung and pulmonary LNs, and its proportion in lungs was significantly lower in the BCG group than in the control group. The BCG group also showed significantly lower mean MNGCs per group than the control group, which may indicate protection, as shown in cattle and macaques [33–35].

*Mycobacterium bovis* BCG is more phylogenetically related to *M. caprae* than the “modern” *M. tuberculosis* strain from which MTBVAC was constructed [36]. Testing MTBVAC in large animal models such as goats, cattle or pigs may be useful because recent studies in Ethiopia and South Africa, where TB is highly prevalent in humans, have described several animal cases of *M. tuberculosis* [37–40]. These studies suggest a complex epidemiology scenario potentially involving zoonotic and anthroponotic TB transmission. In the present study, MTBVAC significantly reduced the number of goats with extra-pulmonary lesions; BCG vaccines showed a similar, albeit nonsignificant, trend featuring gross lesions restricted mainly to the lungs and pulmonary LNs, as previously described in BCG-vaccinated goats [30]. This may be quite relevant for identifying vaccines capable of protecting against severe primary progressive disease in human infants [41]. Nevertheless, 75% of unvaccinated goats had extra-pulmonary lesions, and 66.7% had lesions in their abdominal organs (data not shown). These lesions in the abdomen are difficult to diagnose routinely at the slaughterhouse and may also be related to oral infection [42].

We evaluated the immunogenicity of MTBVAC and BCG vaccines using cellular and humoral techniques before exposure to infected donor goats. MTBVAC-vaccinated animals showed a higher IFN-γ response to PPD-B than BCG-vaccinated ones at 1 month post-vaccination. This is consistent with a previous study in which IFN-γ levels in SO2-vaccinated animals peaked between 1 and 2 months post-vaccination [18]. In the present study, IFN-γ levels remained higher in the MTBVAC group than in the BCG group at 3 months post-vaccination, suggesting long-lasting immunity as described in guinea pigs [43].

The E/C cocktail of ESAT-6 and CFP-10 synthetic antigens, which are absent from *M. bovis* BCG, was developed as a DIVA for IGRA in cattle [44]. The E/C cocktail showed high specificity in BCG-vaccinated cattle, goats and sheep [18, 45–47]. In the present study, however, higher levels of IFN-γ in response to E/C were observed in the MTBVAC group than in the control group at 1 month post-vaccination, and three positive MTBVAC animals were over the cut-off. The MTBVAC and BCG groups showed lower rates of sustained conversion in the IGRA E/C than the control group, especially at T7 and T9. This may reflect sustained *M. tuberculosis* infection, as described in humans after revaccination with BCG [48]. It has been suggested that E/C reactivity may be a biomarker of protection, as observed in C3H mice vaccinated with MTBVAC or with a mutant substrain lacking the *cfp10* and *esat6* genes (MTBVACΔE6C10) [49]. We did not observe a correlation between IFN-γ release after stimulation with E/C and total lesion score at 1 month post-vaccination (T1) and before exposure. A previous experiment in cattle described a positive correlation following exposure to infected donors [50] but we only observed similar results at the end point analysis.

Since MTBVAC and the SO2 prototype contain the RD1 region, which encodes the E/C antigens that are responsible for slight IGRA reactivity in animal trials [18, 49], new biomarkers are needed as DIVA reagents. Alternatively, new cut-offs points should be investigated. Data from the first human trial of MTBVAC showed that the ELISPOT response of samples from vaccinated individuals after E/C stimulation was below the cut-off established for TB infection [49, 51]. A potential solution may be to combine the E/C cocktail with the Rv3615c peptide, shown to act as a DIVA antigen in BGC-vaccinated animals [52]. Although Rv3615c is present in the BCG genome, it cannot be secreted [53]. Adding the Rv3615c peptide to the E/C cocktail increased IGRA sensitivity from 82 to 90% with samples of naturally-sensitised reactor cattle [54]. In our study, 58.3% of infected control animals were E/C reactors at the end of the study, and this proportion increased to 75% when the response to Rv3615c was interpreted in parallel. A similar effect was observed with SIT and SCIT tests in cattle [54].

In the present study, the humoral response in unvaccinated goats was detected later (4 months post-exposure) than cell-mediated response. Herd testing showed higher positivity to P22 ELISA at 2 months after the intradermal tests (T5 and T11) than before these tests; this positivity was probably boosted by the inoculation of intradermal PPDs [55]. Using the P22 ELISA in parallel with cell-mediated techniques allowed detection of all infected goats in the control group at the final test point as reported in previous studies using unvaccinated animals [55].

In conclusion, under a natural tuberculosis infection, all vaccinated animals showed lesions consistent with TB at the end of the study. Nevertheless, both MTBVAC and BCG vaccines proved to be immunogenic and effective in reducing severity of TB pathology caused by *M. caprae*. BCG and MTBVAC were associated with similar gross lesion scores, so further efficacy studies in large animal models evaluating the protection conferred by MTBVAC and BCG vaccines against different MTBC species such as *M. tuberculosis*, *M. bovis* and *M. caprae* are needed in order to elucidate the influence of MTBC species on MTBVAC and BCG efficacy. The E/C peptide cocktail (IGRA) or protein cocktail (intradermal test) proved to be highly specific as DIVA antigens in BCG-vaccinated animals, but less sensitive than PPDs. However, a low number of MTBVAC-vaccinated goats were positive reactors to IGRA stimulated with E/C. The development of new biomarkers used as DIVA reagents would facilitate the potential implantation of MTBVAC in the future.

## Abbreviations

CFU: colony-forming units
DIVA: differentiate infected from vaccinated animals
E/C: ESAT6/CFP10 antigen cocktail
E%: ELISA percentage
IFN-γ: interferon-gamma
IGRA: interferon-gamma release assay
H&E: haematoxylin and eosin
LNs: lymph nodes
MNGCs: multinucleated giant cells
MTBC: *Mycobacterium tuberculosis* complex
OD: optical density
PPD: purified protein derivative
PPD-A: avian purified protein derivative
PPD-B: bovine purified protein derivative
SIT: single intradermal tuberculin
SCIT: single comparative intradermal tuberculin
TB: tuberculosis
TBCL: tuberculosis-compatible lesions
